# A randomised feasibility tolerability study of aminophylline for the prevention of preterm labour

**DOI:** 10.1186/s12884-025-07488-1

**Published:** 2025-03-27

**Authors:** Natasha Singh, Nishel Mohan Shah, Gavin Sooranna, Miriam Bourke, Angela Yulia, Cheryl Battersby, Rachel M. Tribe, Mark R. Johnson

**Affiliations:** 1 Directorate of Women and Children, Department of Metabolism, Digestion and Reproduction, Chelsea and Westminster Hospital London, Imperial College London, London, UK; 2 Department of Metabolism, Digestion and Reproduction, Imperial College, London, UK; 3 Research and Development, University College London Hospitals NHS Foundation Trust, 235 Euston Road, London, NW1 2BU UK; 4 Section of Neonatal Medicine, Department of Medicine, Imperial College London, London, UK; 5 Life Sciences and Medicine, St Thomas’ Hospital Campus, King’s College London, London, UK; 6 Department of Metabolism, Digestion and Reproduction, Faculty of Medicine, Imperial College London, Chelsea & Westminster Hospital, 369 Fulham Road, London, SW10 9NH UK

**Keywords:** Aminophylline, Preterm birth, Progesterone, Preterm prevention

## Abstract

**Background:**

Progesterone is known to maintain uterine quiescence as pregnancy advances. Recently, its efficacy in preventing preterm birth has been questioned prompting a search for an alternative treatment option. Cyclic AMP has been shown *invitro* to act in synergy with progesterone to maintain uterine quiescence.

**Methods:**

We undertook an open label randomised feasibility study to test the hypothesis that the addition of aminophylline to the standard of care (SoC) is acceptable and can be tolerated in pregnant women at high risk of spontaneous preterm labour (sPTL). Women at high risk of sPTL, who met the inclusion criteria were invited to participate and randomised to receive the SoC (progesterone alone, *n* = 33) or treatment with the SoC and aminophylline (*n* = 37). The main outcome measure was to assess the how many women at high-risk of sPTL tolerated and continued to take aminophylline. Data were analysed using Graphpad Prism 8.0c (Graphpad Software, San Diego, CA, USA).

**Results:**

We found that of the addition of aminophylline was well tolerated in 30 of the 33 (91%) of women who continued in the combined arm, without any additional adverse maternal or fetal outcomes. 58% of eligible women agreed to participate in the study. The compliance rate was high at 99.42% +-0.82%. 67% of the women completed the post study questionnaire and all stated their willingness to take aminophylline if it were offered routinely for the prevention of sPTL.

**Conclusions:**

The addition of aminophylline to the SoC is acceptable to women at high-risk of sPTL confirming that a randomised trial of aminophylline to reduce preterm delivery in women at high-risk of PTL is feasible.

**Trial registration:**

Clinical trial gov NCT03152942. Date of full registration: 15/5/2017. https://clinicaltrials.gov/ct2/show/NCT03152942?cond=NCT03152942%26;draw=2%26;rank=1.

**Supplementary Information:**

The online version contains supplementary material available at 10.1186/s12884-025-07488-1.

## Background

Preterm birth (PTB) rates are increasing [1] and accounts for 11% of all pregnancies globally and 8% of births in the UK [2, 3] and is the leading cause of neonatal mortality. In women at high risk of PTB, progesterone (P4) supplementation is used clinically to reduce the risk of preterm labour (PTL) [4–7]. The OPPTIMUM trial, questioned the efficacy of P4 at reducing the risk of spontaneous PTB [8] and Blackwell et al. demonstrated that treatment with injectable 17-α-hydroxyprogesterone caproate did not reduce the risk of PTB among women high risk of PTB before 35 weeks [9]. The recent EPPPIC meta-analysis reported that vaginal progesterone (RR 0.78) and to a lesser extent oral progesterone (RR 0.60) reduced the risk of PTB before 34 weeks in high-risk women [10]. The conflicting evidence has prompted a call for research to be directed to alternative treatment options that may include adjunctive therapy to improve the effectiveness of P4 [11].

An alternative approach is to increase the intracellular levels of cyclic adenosine monophosphate (cAMP) in myometrial smooth muscle cells. cAMP is a potent smooth muscle relaxant with anti-inflammatory properties, which maintains myometrial quiescence during pregnancy [12] and is up-regulated by many endogenous pro-pregnancy hormones including relaxin, human chorionic gonadotropin and calcitonin gene-related peptide [13]. Therapeutically, increases in cAMP are commonly achieved by giving beta-adrenergic agonists like ritodrine or terbutaline. However, these drugs have severe cardiovascular side effects preventing their use in cases of PTL. Other approaches to increasing myometrial cAMP have been explored in cell, tissue and animal-based studies by inhibiting the metabolism of cAMP using a phosphodiesterase inhibitor. Our data suggest that this is a highly effective approach with fewer side effects [14]. We used aminophylline, a non-specific phosphodiesterase inhibitor, to increase intra-cellular cAMP, and showed that it reduces the response to IL-1b, and potentiates P4 action [15], inhibits myometrial strip contractility and potentiates the inhibitory action of P4 in a mouse model of PTL [14]. Further, animal and human data suggest that aminophylline improves perinatal outcomes, reducing the risk of respiratory distress syndrome, intraventricular haemorrhage [16–18] and bronchopulmonary dysplasia in extremely preterm infants [19]. Despite these beneficial effects, no formal studies have assessed the impact of maternally administered aminophylline on pregnancy outcomes.

Aminophylline is available as a modified release preparation called Phyllocontin Continus^®^ which is given orally and contains aminophylline hydrate 225 mg. The medication is typically used for both children and adults to treat asthma and is also available in an intravenous preparation. The recommended oral dose in adults is 225 mg twice a day, which is increased after one week to two tablets per day [20, 21]. Aminophylline is rapidly metabolised to theophylline and peak serum levels are reached after 1.5–2 h of treatment, and toxic levels for humans are above 20ng/ml. Serum levels of theophylline are at or below 20ng/ml are associated with mild side effects such as nausea, vomiting, headache and insomnia. Side effects such as diarrhoea, irritability, restlessness, fine tremors and transient diuresis can also be reported at therapeutic levels between 10-20ng/ml [22]. Theophylline peak levels occur 1 to 3 h after oral ingestion of aminophylline and considered safe to use if breastfeeding is required [23]. Our objective was to undertake an open-label feasibility study to investigate the acceptability of oral aminophylline in women at high risk of PTB.

## Methods

### Study design

**Fig. 1:**
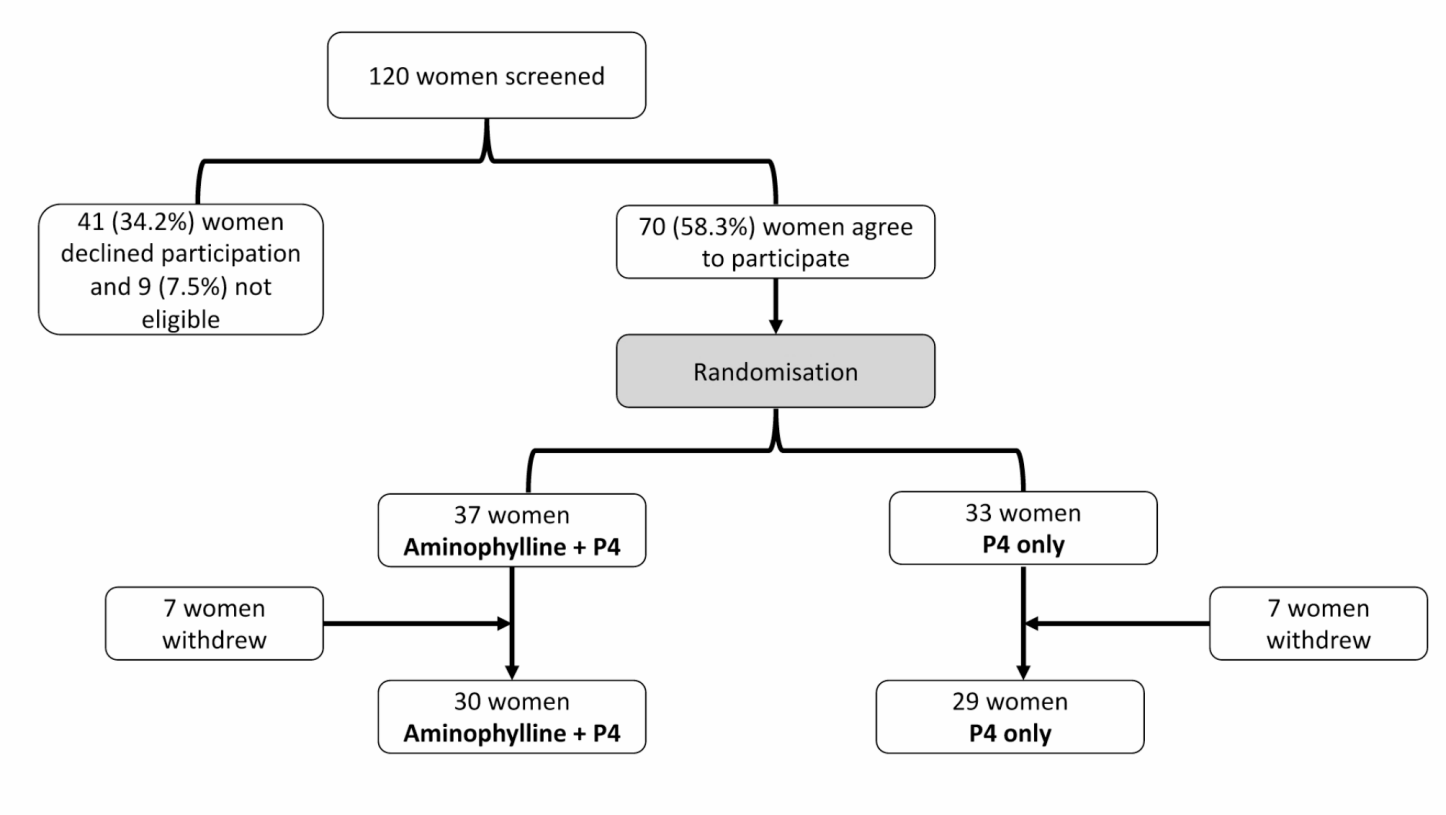
CONSORT flow diagram for the trial showing participant flow through each stage of the feasibility randomised trial (enrolment, intervention allocation). P4– progesterone

The study was an open label feasibility randomised study in a single centre. The study was approved by the East of England, Cambridgeshire and Hertfordshire Research Ethics Committee 16/EE/0299. Women attending the preterm birth surveillance clinic during October 2017 to March 2021 were screened for eligibility by the trial research midwife. Those who wished to participate and met the inclusion criteria were randomised to receive standard of care (SoC) alone or SoC with aminophylline. During the study, the trial management team, held monthly meetings to review the conduct and safety of the study and this meeting was also attended by the study patient and public involvement (PPI) lead. The study PPI group chaired by the PPI lead, held four meetings during the study and all participants completed an end of study questionnaire. Figure 1 summarises the CONSORT diagram.

### Study population

Pregnant women who attended the prematurity clinic at the Chelsea and Westminster Hospital were assessed for study eligibility. All women with a singleton pregnancy were approached to participate if they were between 13 and 20 weeks of gestation, had a previous mid trimester loss (*n* = 19), previous spontaneous PTB (*n* = 28), short cervical length (*n* = 2), history indicated cervical suture (*n* = 18), ultrasound indicated cervical suture (*n* = 21), previous cervical surgery (*n* = 5), and previous mid trimester medical termination of pregnancy (*n* = 1, Table 1). Women were excluded if they reported any history of intolerance to aminophylline or if there were on any medications which would interact with the study Investigational Medicinal Product (IMP). The inclusion and exclusion criteria used are summarised in Table S1.

**Table 1:** Demographics of women and pregnancy outcomes in the study (excluding the withdrawals)

	SoC and aminophylline treatment (*n* = 30)				SoC (*n*=-29)			
*Age (years)	36 ±4.0				33 ±5.1			
*BMI	24.5+-6.1				24.0 ±5.0			
Smoking status	0				0			
*GA at baseline	16.6 weeks ±2.2				15.4±2.7			
Preterm birth risk factors								
Short cervix in pregnancy	0				2 (6.9%)			
Second trimester loss	11 (36.7%)				8 (27.6%)			
Spontaneous PTB in prev pregnancy	14 (46.7%)				14 (48.3%)			
MTOP second trimester	1 (3.3%)				0			
Cervical treatment	3 (10%)				2 (6.9%)			
Cervical suture in previous pregnancy	3 (10%)				2 (6.9%)			
Ethnicity								
Black (British, African, Caribbean)	4 (13.3%)				2 (6.9%)			
Indian	3 (10%)				1 (3.4%)			
Asian	2 (6.7%)				0			
Middle eastern	1 (3.3%)							
White	8 (26.7%)				10 (34.5%)			
Other/mixed	5 (16.7%)							
Cervical suture during pregnancy								
History indicated suture	9 (30%)				9 (31%)			
Ultrasound indicated cervical suture	12 (40%)				9 (31%)			
Transabdominal suture	1				0			
Maternal outcomes								
Blood loss at birth	425mls±394.9mls				400mls±260.0mls			
GDM	4 (13.3%)				3 (10.3%)			
PET	0				2(6.9%)			
Fetal outcomes								
Admission to NICU	5 (16.7%)				3 (10.3%)			
Extreme Prematurity (< 26 weeks)	0				1 (3.4%)			
26 weeks to 31 weeks	1 (3.3%)				0			
32 weeks to 36 weeks	4 (13.3%)				4 (13.8%)			
Fetal abnormality	1 (3.3%)				2 (6.9%)			
*Length of latency of pregnancy (weeks)	11.07±9.06				8.36±7.22			
Side effect and tolerability profile								
	A^δ^	B^ε^	C^γ^	D^λ^	A^δ^	B^ε^	C^γ^	D^λ^
No of women	24				20			
Stomach upset		3 (12.5%)		21 (87.5%)				20 (100%)
Diarrhoea		4 (16.7%)	1 (4.2%)	19 (79.2%)		1 (5%)		19 (95%)
Restlessness		5 (20.8%)	5 (20.8%)	16 (66.7%)		1 (5%)		19 (95%)
Headaches		3 (12.5%)	3(12.5%)	18 (75%)		2 (10%)		18 (90%)
Increased heart rate	1 (4.2%)	7 (29.2%)	4 (16.7%)	12 (50%)		1 (5%)		19 (95%)
Skin rashes	1 (4.2%)	1 (4.2%)	1 (4.2%)	21 (87.5%)				20 (100%)
Other**	Mood swings *n* = 1 (4.2%) Dry mouth *n* = 1 (4.2%) Occasional dizziness and nausea *n* = 1 (4.2%)				Severe constipation *n* = 1 (5%) Depression and anxiety *n* = 2 (10%)			

*median±standard deviation

^δ^ Strongly agree and would not like to repeat treatment

^ε^ Agree but would still repeat the treatment

^γ^ Neither agree or disagree

^λ^Disagree

### Study intervention

**Fig. 2:**
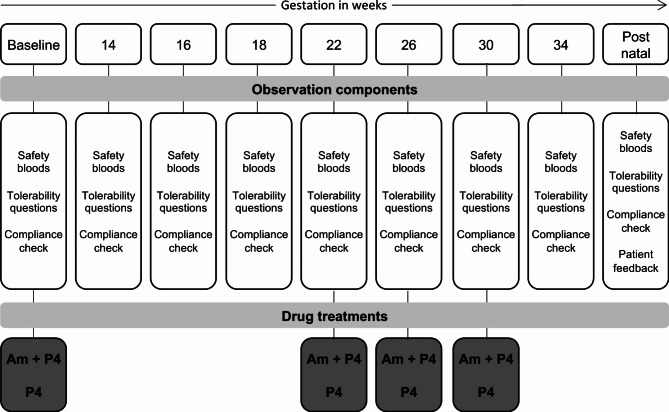
Shows the study visits, observational components for each participant at each visit, and at which timepoint drug treatments were issued to participants. IMPs were either Aminophylline and vaginal progesterone (Am + P4) or vaginal progesterone only (P4)

Standard of care (SoC) for high-risk women comprised a vaginal P4 with or without a cervical suture also inserted. Once recruited to our study, women were randomised to receive either receive SoC alone or with aminophylline. Aminophylline was issued from commercial supplies by Chelsea and Westminster Hospital pharmacy. Randomisation was done by the Chief Research Pharmacist using sealed envelopes. All IMPs were labelled for clinical trial use at the point of dispensing by the Chelsea & Westminster Hospital pharmacy department. Following randomisation, a clinical trial specific prescription was generated detailing the number of tablets necessary and instructions for use. This was based on a standard prescription template which was reviewed by the clinical trial monitor prior to recruitment. Prescriptions were fulfilled by the pharmacy and supplied to allow for compliance to be continually checked and to avoid wastage. The trial research midwife assured that the women received the study prescription and witnessed the first study drug dosing. Study visits, pharmacy supply, study questionnaire and observational components of the study comprised the following (Fig. 2):

1. Supply of the IMP was provided from baseline to week 22, week 22 to week 26, week 26 to week 30 and week 30 to week 34 of the pregnancy.
2. At the baseline visit, safety bloods (liver function, creatinine, urea, electrolytes, full blood count and clotting) were taken and concomitant medications checked.
3. At subsequent visits, safety bloods, tolerability, compliance with medications were checked at visits at 14, 16, 18, 22, 26, 30 and 34 weeks of pregnancy with a +/- 5-day window.
4. At 6 weeks after delivery, (+/- 1-week window) a final study visit was conducted, and a participant feedback questionnaire was completed.
5. Post randomisation the following medications were discouraged unless there is a clear and recognised indication: (i) Acyclovir, calcium channel blockers, cimetidine, erythromycin, clarithromycin, corticosteroids, and benzodiazepine carbamazepine and beta-sympathomimetics as they interact with aminophylline, and some can potentiate hypokalaemia.
6. No concomitant medications (prescription, over the counter or herbal) were administered during the study unless they were approved/prescribed by the Investigator for treatment of specific clinical events. All concomitant therapies were recorded on the case report form (CRF).

### Study outcomes and data recording

Outcomes:

1. Primary outcome of the study was the number of women who withdrew due to side effects.
2. Secondary outcomes recorded were the number of women who:
  I. Were eligible to be recruited.
  II. Those who were willing to participate.
  III. Those who completed the study.
  IV. In those who did not complete the study, the reasons for not completing.

Additional data recorded: We also recorded the demographic data for the women, weekly caffeine consumption and both pregnancy, and neonatal outcomes.

Adverse reporting: Adverse outcomes (for example PTB and admission to neonatal unit) were anticipated due to the study population and all admissions to hospital were regarded as an adverse outcome and reported via the CRF. All admissions and pregnancy losses were reviewed by the trial monitoring team which included the chief investigator and principal investigator to determine if outcome was directly related to the study.

### Sample size and statistical analysis

As this was a feasibility study a full power calculation was not performed. Data were analysed using Graphpad Prism 8.0c (Graphpad Software, San Diego, CA, USA). P values were two tailed and significance was defined as *P* < 0.05. For continuous data, data distribution was assessed using a Shapiro-Wilk test. Normally distributed data were analysed using a Student’s t-test for the two groups. Non-parametric data were analysed using the Kolmogorov-Smirnov test for the two groups.

## Results

One hundred twenty women were screened and approached to participate in the study. 41 women declined participation (Table S2 summarises the reasons for the declining) and 9 did not meet the inclusion criteria. Of the 70 women who met the inclusion criteria and agreed to participate, 33 were randomised to SoC alone and 37 women were randomised to receive SoC and oral Aminophylline 225 mg twice a day. The demographics and baseline characteristics of the women in the study are summarised in Table 1. There was no statistically significant difference in the age, BMI, and gestation at recruitment between the two treatment groups. All the women in the study reported that they did not use caffeine throughout the study.

The primary outcome showed that 91% of women were able to tolerate aminophylline. Four women withdrew from the SoC arm, all for non-treatment related reasons, and 7 women withdrew in the SoC and aminophylline arm (3 for treatment related side effects and 4 for other reasons; Table 2). The median duration of aminophylline treatment before withdrawal was 5 days with range 1 to 39 days. All women who withdrew had a live birth at term.

**Table 2:** Reasons for withdrawal

Reason for withdrawal	Combined*	P4 only*
Side effects	3	0
Patient related	2	3
Chief investigator’s decision to withdraw due to multiple DNA	2	1

### Compliance outcomes

#### Overall compliance

Compliance for each participant was calculated via pack returns, patient diaries, and patient self-reports and reported as a proportion of the total doses of study medication used divided by the expected doses. Adequate compliance was taken as 80% of prescribed medication. For aminophylline, the median compliance was 99.42% +-0.82%.

#### Tolerability and side effects

Tolerability questionnaires were completed at study visits 14, 16, 18, 22, 26, 30 and 34 weeks of pregnancy. The participants were asked about the side effects that they experienced based on the 5 point Likert scale and whether it would affect a decision to take the treatment in the future [24]. Our findings are summarised in Table 1. In the SoC with aminophylline arm, more women reported side effects higher on the likert scale such as gastrointestinal upset, palpitations, headaches and skin rashes when compared to the SoC arm. The participants were asked if despite the side effects, they would accept the treatment in the future. Only two women in the aminophylline arm reported that they would not like to repeat the treatment in the future because of the palpitations and a skin rash they experienced respectively. In the SoC arm, two women reported depression/anxiety and one reported severe constipation.

### Patient and public involvement outcomes

At the end of the study at 6 weeks postnatal, 63 participants who remained in the study, were sent a feedback questionnaire and 42 completed questionaries were returned (67% compliance with the questionnaire). 23 women in aminophylline arm and 19 in the SoC arm returned the feedback questionnaire (Table 3). In both arms of the study, 100% of the women who responded stated that they would accept the treatment in another study or if it became normal practice. However, it is important to note that the 3 women who did not tolerate Amniophylline did not complete the questionnaire.

**Table 3:** Participant feedback during the study

	Combined (*n* = 23)	P4 (*n* = 19)
How did you feel about the treatment you were allocated?		
Extremely satisfied	18	16
Fairly dissatisfied	1	1
Fairly satisfied	4	2
Extremely dissatisfied	0	0
How would you feel if the treatment you were allocated to, became normal practice?		
Disappointed	0	0
Not sure	0	0
Pleased	23	19
Would you recommend a friend or relative to participate in the trial?		
Probably not	0	0
Not sure	0	0
Probably	2	1
Yes	8	4
Definitely yes	13	14

Regarding feedback on the overall participation of the study, 27 women said they would ‘definitely recommend’ a friend or relative to participate in the study. Thirty-four women reported that they were ‘extremely satisfied’ with the treatment arm they were allocated and only 2 reported that they were ‘fairly dissatisfied’. All the women reported that they were given enough information during the study.

### Maternal outcomes

There was no statistical difference in the rates of gestational diabetes (GDM) or pre-eclampsia (PET), and the volume of blood loss at birth between the two treatment groups. In the SoC arm, there were 3 admissions for threatened PTL and one woman gave birth at 24 weeks. In the aminophylline group, there were 3 admissions for threatened sPTL and one woman gave birth at 33 weeks. There was one maternal admission to ITU for COVID-19 related reasons. Maternal outcomes are summarised in Table 1.

### Fetal outcomes (live births, admission to NICU)

Fifty-three (76.7%) women in the study had a previous pregnancy. In the SoC arm, 15 women had a previous live birth and 9 had previous pregnancy loss. In the aminophylline arm, 15 women had a previous live birth and 14 had a previous pregnancy loss.

At the end of the study treatment, 28 women in the aminophylline arm had a live birth and two women had a pregnancy loss (at 19 weeks and 22 weeks of gestation). In the SoC arm, all 29 women had a live birth. There was one extreme preterm birth at 24 weeks in the SoC arm. Six women were in their first pregnancy (5 in SoC arm and 1 in aminophylline arm), and they all had a live birth. There were 5 cases of admissions to NICU in the aminophylline arm, related mainly to prematurity and the need for respiratory support. In the SoC arm, there were 3 admissions to NICU, two cases were for prematurity (24 weeks and 34 weeks) and the other at 38 weeks for reasons unrelated to the study medication since treatment with progesterone stopped at 34 weeks of gestation. The median stay for babies admitted to the neonatal unit was 17days+-21.7 days. Fetal outcomes are summarised in Table 1. At the end of the study, one of the babies born at 24 weeks was still an inpatient on the neonatal unit.

### Outcomes on length of latency of pregnancy

We calculated the length of latency of the pregnancy among birth study groups. In.

SoC with aminophylline arm the length of latency was 10.05 weeks ± 7.9 weeks (*n* = 28) and in the SoC arm it was 9.63 weeks ± 7.2 (*n* = 24) weeks but this was not statistically significant (Figure S1).

### Serious adverse events reported during the study

All admissions and pregnancy losses were treated as serious adverse events (SAEs) and reported and reviewed by the trial management team. In the aminophylline arm, there were 8 reported SAE and two in the SoC arm, and these are summarised in Table S3. None of the SAEs that were considered to be likely related to the study intervention were associated with any significant adverse maternal or neonatal outcomes. There was one intrauterine demise at 22 weeks in the SoC with aminophylline arm. This patient had a transabdominal suture, and the cause of death was due to sepsis. One woman in the SoC with aminophylline arm presented at 19 weeks with abdominal pain and vaginal bleeding and miscarried. There were no pregnancy losses in the SoC arm. Two women in the aminophylline arm were admitted for COVID-19 infection.

## Discussion

### Main findings

Our main finding was that the addition of oral Aminophylline to SoC was well tolerated by women at high-risk of sPTB. Compared to SoC, the addition of oral aminophylline was not associated with any significant adverse maternal or neonatal outcomes. The women reported that if aminophylline were to be part of SoC they would accept the treatment.

### Strength and limitation

A major strength is that is a randomised study among women at high risk of sPTL. Furthermore, the feasibility study had a recruitment rate of 58%. However, the limitation of this study is that it was a feasibility study and therefore not powered to show a reduction in preterm birth < 35 weeks.

### Interpretation

Collectively, previous data suggest that a cAMP agonist administered alone or in combination with progesterone supplementation might delay, preterm birth and improve perinatal outcomes [14, 15]. The current study was a feasibility study and was not powered to investigate any other perinatal outcomes. There was no difference in the postnatal outcomes which in some cases may be because we stopped the aminophylline at 34 weeks and 25 births occurred after this point. For this reason, we plan to continue the aminophylline until 36 weeks in the future study. Furthermore, given that aminophylline and caffeine are methylxanthines and have similar effects, the women in the study did not ingest caffeine and we are confident that caffeine consumption was not a confounder of the observations.

Preterm birth is not a single entity, rather it has multiple aetiologies and presenting with different phenotypes [25]. This may account for the conflicting response to P4 among women at an individual level. Our group has shown that P4 has anti-inflammatory effects and that these can be enhanced by the addition of a cAMP agonist in primary myometrial cells and in a mouse model of inflammation-induced PTL [14]. Equally, we failed to show any additive effect on in vitro myometrial strip contractility [15]. These data suggest that cAMP with progesterone may be most effective against idiopathic PTL, where inflammation drives the onset of labour.

This is a feasibility study not powered to show any difference in the outcome of pregnancy. In both arms of the study, 1 birth occurred between 24 and 34 weeks. Previous studies would suggest that in this high-risk population, 16–20% of women treated with progesterone would have been expected to deliver before 34 weeks [5, 8]. Had we seen a similar result in the current study, we would have expected 5–7 women to have delivered in the SoC arm as opposed to the 1 case that we saw. The unexpectedly low rates of preterm birth in the SoC arm limits our ability to perform any useful power calculation to inform a larger study of SoC with aminophylline vs. SoC alone.

## Conclusions

This feasibility study showed that aminophylline is well tolerated and that subjects were highly compliant supporting its acceptability in this high-risk population and therefore supports the further investigation of the oral aminophylline in a double-blinded randomised controlled trial as a potential new treatment option for the prevention of sPTL and improvement in perinatal outcomes.

## Electronic supplementary material

Below is the link to the electronic supplementary material.

Supplementary Material 1

Supplementary Material 2

Supplementary Material 3

Supplementary Material 4

## Data Availability

All data generated or analysed during this study are included in this published article.

## Abbreviations

cAMP: cyclic adenosine monophosphate
CRF: Case report form
IMP: Investigational Medicinal Product
PPI: Patient and public involvement
PTB: Preterm birth
PTL: Preterm labour
P4: Progesterone
sPTL: spontaneous preterm labour
SoC: Standard of care
