# Supplementary material for: A randomised feasibility tolerability study of aminophylline for the prevention of preterm labour
Source: BMC Pregnancy Childbirth. 2025 Mar 27;25:357. doi: 10.1186/s12884-025-07488-1 (PMC11948830; doi:10.1186/s12884-025-07488-1)
Supplement: Supplementary file 2 — Supplementary Material 2 [file 12884_2025_7488_MOESM2_ESM.docx]

Table S1 Inclusion and exclusion criteria

| Inclusion | Exclusion |
| --- | --- |
| Pregnant women between 13 and 20 weeks of gestation. | Known sensitivity, contraindication or intolerance to P4 (history of liver tumours, severe liver impairment, general or breast cancer, severe arterial disease, undiagnosed vaginal bleeding, acute porphyria, history during pregnancy of idiopathic jaundice, severe pruritus or pemphigoid gestationis). |
| Singleton pregnancy | Known sensitivity, contraindication or intolerance to aminophylline (hypokalaemia, pre-existing cardiac arrhythmias, epilepsy, hyperparathyroidism, peptic ulcer disease) |
| Intact fetal membranes at the time of recruitment. | Suspected or proven rupture of the fetal membranes at the time of recruitment. |
| The ability to understand and sign a written informed consent form, prior to participation in any screening procedures and must be willing to comply with all study requirements. | Participants taking prescribed medications known to interact with P4 (e.g. Bromocriptine, carbamazepine, diazepam, lorazepam and temazepam, insulin) or aminophylline at screening. |
| Women must be aged 18 years or older. | Participants taking prescribed medications that are known to increase Aminophylline plasma concentrations: acyclovir, calcium channel blockers, cimetidine, erythromycin, clarithromycin, corticosteroids and benzodiazepine, carbamazepine, beta-sympathomimetics at screening. |
| Obstetric history of one or more of the following:   1. Previous mid-trimester loss (14-26 weeks) 2. Previous preterm delivery (<37 Weeks) 3. Women with a history of indicated cervical suture 4. Short cervical length(≤25mm) on ultrasound at 13-20+0 weeks gestation, with or without cervical suture and with or without a positive fetal fibronectin | Evidence of maternal infection or sepsis (maternal temperature ≥37.5oC, increased inflammatory markers – WBC, CRP). |
|  | Multiple pregnancy. |
|  | Known significant congenital structural or chromosomal fetal anomaly. |
|  | Maternal pathologies in which preterm termination of pregnancy is required. |
|  | Pre-eclampsia or severe hypertension. |
|  | Participants who smoke, drink alcohol or take recreational drugs. |
