# Supplementary material for: A randomised feasibility tolerability study of aminophylline for the prevention of preterm labour
Source: BMC Pregnancy Childbirth. 2025 Mar 27;25:357. doi: 10.1186/s12884-025-07488-1 (PMC11948830; doi:10.1186/s12884-025-07488-1)
Supplement: Supplementary file 3 — Supplementary Material 3 [file 12884_2025_7488_MOESM3_ESM.docx]

Table S2 Reasons women declined participation

| Reason for declining participation | | Number of women |
| --- | --- | --- |
| 1 | Doesn't want to take part in any research | 14 |
| 2 | Doesn't want to take part in tolerability study | 3 |
| 3 | Doesn't want to take extra medication | 10 |
| 4 | Doesn't want extra appointments | 5 |
| Combination of reasons for declining | |  |
| 2,3 |  | 2 |
| 3,4 |  | 7 |
