# Supplementary material for: A randomised feasibility tolerability study of aminophylline for the prevention of preterm labour
Source: BMC Pregnancy Childbirth. 2025 Mar 27;25:357. doi: 10.1186/s12884-025-07488-1 (PMC11948830; doi:10.1186/s12884-025-07488-1)
Supplement: Supplementary file 4 — Supplementary Material 4 [file 12884_2025_7488_MOESM4_ESM.docx]

Table S3 Serious Adverse Events reported

| Combined treatment with Aminophylline and progesterone | Progesterone only treatment |
| --- | --- |
| Threatened preterm labour | Pre-eclampsia |
| Diarrhoea and vomiting | Threatened preterm labour |
| Intrauterine demise at 22 weeks | Preterm labour |
| Vaginal bleeding |  |
| 31 weeks rupture of membranes and contracting |  |
| Hyperemesis gravidarum |  |
| COVID-19 symptoms |  |
| Miscarriage |  |
