# Supplementary material for: A randomised feasibility tolerability study of aminophylline for the prevention of preterm labour
Source: BMC Pregnancy Childbirth. 2025 Mar 27;25:357. doi: 10.1186/s12884-025-07488-1 (PMC11948830; doi:10.1186/s12884-025-07488-1)

Figure S1 Latency of pregnancy

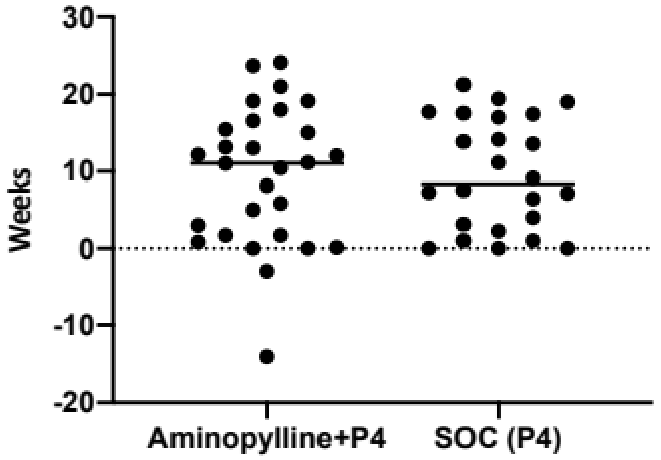

Supplement: Supplementary file 4 [file 12884_2025_7488_MOESM1_ESM.docx]
